# N-Linked Glycans Are Assembled on Highly Reduced Dolichol Phosphate Carriers in the Hyperthermophilic Archaea *Pyrococcus furiosus*

**DOI:** 10.1371/journal.pone.0130482

**Published:** 2015-06-22

**Authors:** Michelle M. Chang, Barbara Imperiali, Jerry Eichler, Ziqiang Guan

**Affiliations:** 1 Departments of Biology and Chemistry, Massachusetts Institute of Technology, Cambridge, MA 02139, United States of America; 2 Department of Life Sciences, Ben Gurion University, Beersheva 84105, Israel; 3 Department of Biochemistry, Duke University Medical Center, Durham NC 27710, United States of America; Weizmann Institute of Science, ISRAEL

## Abstract

In all three domains of life, N-glycosylation begins with the assembly of glycans on phosphorylated polyisoprenoid carriers. Like eukaryotes, archaea also utilize phosphorylated dolichol for this role, yet whereas the assembled oligosaccharide is transferred to target proteins from dolichol pyrophosphate in eukaryotes, archaeal N-linked glycans characterized to date are derived from a dolichol monophosphate carrier, apart from a single example. In this study, glycan-charged dolichol phosphate from the hyperthermophile *Pyrococcus furiosus* was identified and structurally characterized. Normal and reverse phase liquid chromatography-electrospray ionization mass spectrometry revealed the existence of dolichol phosphate charged with the heptasaccharide recently described in *in vitro* studies of N-glycosylation on this species. As with other described archaeal dolichol phosphates, the α- and ω-terminal isoprene subunits of the *P*. *furiosus* lipid are saturated, in contrast to eukaryal phosphodolichols that present only a saturated α-position isoprene subunit. Interestingly, an additional 1-4 of the 12-14 isoprene subunits comprising *P*. *furiosus* dolichol phosphate are saturated, making this lipid not only the longest archaeal dolichol phosphate described to date but also the most highly saturated.

## Introduction

The polyisoprenols are a family of hydrophobic polymers comprising up to more than 100 isoprene subunits bearing a terminal hydroxyl group at the α-terminus of the molecule [1–3]. The two main groups of polyisoprenoids, polyprenols and dolichols, can be distinguished by the presence of an unsaturated (polyprenols) or saturated (dolichols) α-isoprene subunit. While various biological roles have been suggested for the polyisoprenoid alcohols, these remain to be clearly delineated [1–5]. On the other hand, phosphorylated polyprenols and dolichols are well established as central players in N-glycosylation, namely the covalent linkage of glycans to select asparagine residues of target proteins [6–8].

In N-glycosylation, the glycan moiety is initially assembled on a phosphorylated polyisoprenol, from where it is transferred to the protein target by the actions of an oligosaccharyltransferase (OST). Whereas bacterial N-linked glycosylation generally relies on polyprenol phosphate species, such as C_55_ undecaprenol phosphate (UndP), as the glycan carrier, eukaryotes and archaea instead rely on phosphorylated dolichols for this purpose [6–8]. Various traits, however, serve to distinguish between the phosphorylated dolichols used in eukaryal and archaeal N-glycosylation. In eukaryal N-glycosylation, dolichols of various lengths are found, with C_80_, C_90_ and C_95_ phosphorylated dolichol predominating in yeast, rat and humans, respectively [1]. Archaeal N-glycosylation also involves dolichol species of multiple lengths, although these tend to be shorter than their eukaryal counterparts, containing only 8–12 five-carbon isoprene units [9–13]. In addition, whereas a phosphosugar is added to dolichol phosphate (DolP) in the first step of the eukaryotic pathway to generate a dolichol pyrophosphate (DolPP)-bound oligosaccharide, archaeal N-glycosylation assembles oligosaccharides on DolP (although in the haloarchaeon *Halobacterium salinarum*, DolP and DolPP have both been proposed to serve this role) [8,14]. Most strikingly, phosphorylated dolichols in eukaryotes and archaea differ in their degrees of saturation. While phosphorylated dolichols contain a saturated α-isoprene unit in organisms belonging to each domain, archaeal DolP also contains a saturated ω-isoprene unit [10,11,13].

In research designed to further our understanding of archaeal N-glycosylation, the OST from *Pyrococcus furiosus*, a hyperthermophilic archaeon isolated from a marine solfatara off the coast of southern Italy that grows optimally at 100°C [15], was addressed. In these studies, *in vitro* enzymatic experiments showed that upon incubation with an extracted pool of lipid-linked oligosaccharides and the full-length OST, an added hexapeptide containing an asparagine-based N-glycosylation signal (a ‘sequon’) was modified by a novel heptasaccharide of recently defined composition [16–18]. Although the glycan-bearing lipid moiety was not characterized in these studies, it was assumed to be DolPP [18].

In the present report, liquid chromatography-electrospray ionization mass spectrometry (LC-ESI MS) was employed to structurally characterize the lipid species bearing the complete glycan recruited in *P*. *furiosus* N-glycosylation, as well as to verify the composition of this heptasaccharide.

## Materials and Methods

### *P*. *furiosus* lipid extraction

*P*. *furiosus* DSM 3638 cells were a gift from Prof. Michael Adams (University of Georgia) [15]. The cell pellet (11 g) was added to a 250 ml round bottom flask and extracted with 2:1:0.8 methanol/chloroform/pellet (50 ml, total volume) for 24 h, stirring at room temperature. The mixture was vacuum-filtered through a Büchner funnel and three filter papers (Whatman Grade 1) and the filtrate was dried down using a rotary evaporator at 30°C. The dried lipid extract (52.3 mg) was stored at -20°C until further analyzed.

### Normal phase (NP)LC-ESI MS

NPLC-ESI MS of the *P*. *furiosus* lipid extract was performed using an Agilent 1200 Quaternary LC system coupled to a high resolution TripleTOF5600 mass spectrometer (Applied Biosystem, Foster City, CA). An Ascentis Si HPLC column (5 μm, 25 cm × 2.1 mm) was used. Mobile phase A consisted of chloroform/methanol/aqueous ammonium hydroxide (800:195:5, v/v/v). Mobile phase B consisted of chloroform/methanol/water/aqueous ammonium hydroxide (600:340:50:5, v/v/v/v). Mobile phase C consisted of chloroform/methanol/water/aqueous ammonium hydroxide (450:450:95:5, v/v/v/v).

The elution program consisted of the following: 100% mobile phase A was held isocratically for 2 min and then linearly increased to 100% mobile phase B over 14 min and held at 100% B for 11 min. The LC gradient was then changed to 100% mobile phase C over 3 min and held at 100% C for 3 min, then returned to 100% A over 0.5 min and held at 100% A for 5 min. The LC eluent (with a total flow rate of 300 μl/min) was introduced into the ESI source of the mass spectrometer. Instrument settings for negative ion ESI/MS and MS/MS analysis of lipid species were as follows: Ion spray voltage (IS) = -4500 V; Curtain gas (CUR) = 20 psi; Ion source gas 1 (GS1) = 20 psi; De-clustering potential (DP) = -55 V; Focusing potential (FP) = -150 V. The MS/MS analysis used nitrogen as the collision gas. Data acquisition and analysis were performed using the Analyst TF1.5 software (Applied Biosystem, Foster City, CA).

### Reverse phase (RP)LC-ESI/MS

RPLC-ESI/MS of the *P*. *furiosus* lipid extract was performed using a Shimadzu LC system (comprising a solvent degasser, two LC-10A pumps and a SCL-10A system controller) coupled to a TripleTOF5600 mass spectrometer (Applied Biosystems, Foster City, CA). LC was operated at a flow rate of 200 μl/min with a linear gradient as follows: 100% of mobile phase A was held isocratically for 2 min and then linearly increased to 100% mobile phase B over 14 min and held at 100% B for 4 min. Mobile phase A consisted of methanol/acetonitrile/aqueous 1 mM ammonium acetate (60/20/20, v/v/v). Mobile phase B consisted of 100% ethanol containing 1 mM ammonium acetate. A Zorbax SB-C8 reversed-phase column (5 μm, 2.1 x 50 mm) was obtained from Agilent (Palo Alto, CA). The MS operating conditions were as described above.

## Results

### *P*. *furiosus* DolP is charged with a heptasaccharide and its precursors

In several archaea, including *Hbt*. *salinarum* [14] and *Sulfolobus acidocaldarius* [12], DolP is charged with the same glycans (or their precursors) as those N-linked to glycoproteins in these organisms. In other instances, such as *Haloferax volcanii* [11] and *Methanococcus voltae* [19], evidence for the delivery of oligosaccharides or single sugars from DolP carriers to target Asn residues has been provided. Although *in vitro* efforts revealed that lipid-linked oligosaccharides are required for *P*. *furiosus* N-glycosylation, the identity of the lipid carrier was not defined [16–18]. To thus determine the nature of this heptasaccharide-charged lipid carrier in *P*. *furiosus*, RPLC-ESI MS was performed.

**Fig 1:**
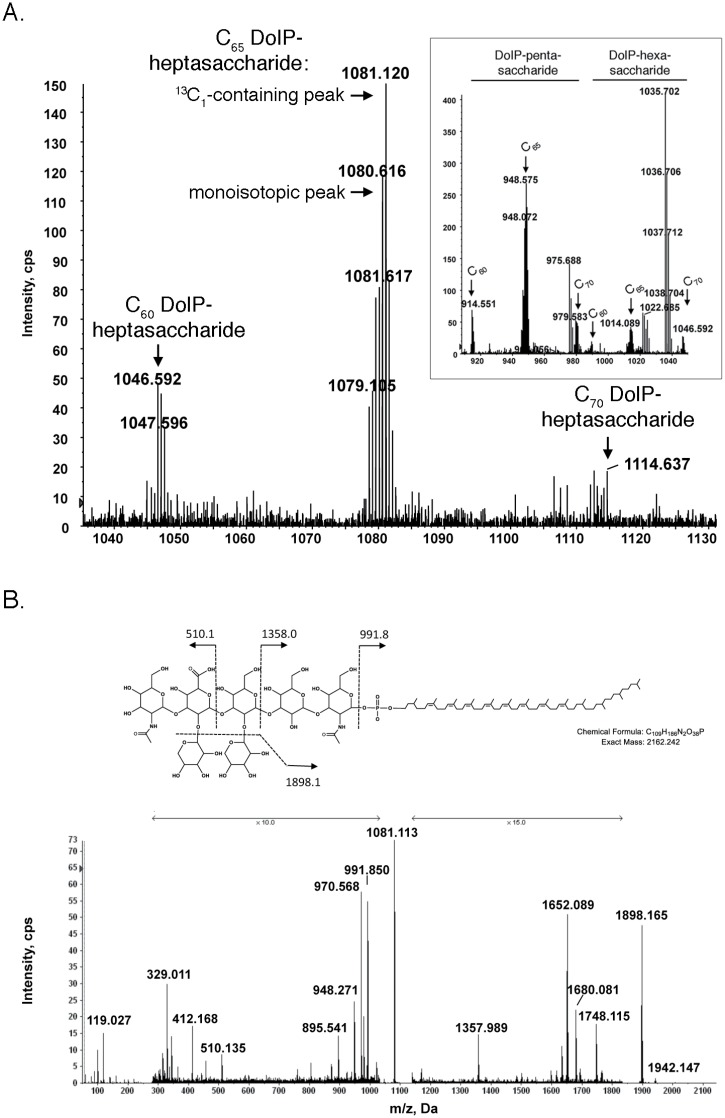
*P*. *furiosus* contains heptasaccharide-charged DolP. A. The mass spectrum of the RPLC fraction with a retention time of 8.5–9.5 min contains [M-2H]^2-^ peaks of *m/z* 1046.592, 1081.120 and 1114.637, corresponding to heptasaccharide-charged C_60_, C_65_ and C_70_ DolP, respectively. The ^13^C_1_-containing isotopic and monoisotopic [M-2H]^2-^ peaks of heptasaccharide-charged C_65_ DolP are indicated. The inset shows [M-2H]^2-^ peaks of *m/z* 914.551, 948.575 and 979.583, corresponding to C_60_, C_65_ and C_70_ DolP attached to the pentasaccharide precursor of the complete heptasaccharide, as well as [M-2H]^2-^ peaks of *m/z* 980.585, 1014.089 and 1048.116, corresponding to C_60_, C_65_ and C_70_ DolP attached to the hexasaccharide precursor of the same heptasaccharide. B. The chemical structure (based on the N-linked glycan) and MS/MS fragmentation scheme are shown in the panel top. The MS/MS spectrum of the [M-2H]^2-^ peak of *m/z* 1081.12 corresponding to heptasaccharide-charged C_65_ DolP is presented in the panel bottom. The arrows indicating x10 and x15 reflect magnification of the ion peaks in the corresponding region of *m/z* values on the spectrum.

When the MS profile of the RPLC fraction with a retention time of 8.5–9.5 min was examined, a doubly de-protonated [M-2H]^2-^ ion peak of *m/z* 1081.120 was observed (Fig 1A). The mass of this ion (observed mass 2164.256 Da) matches with C_65_ DolP linked to the heptasaccharide that *P*. *furiosus* AglB was previously shown to add to sequon-containing peptides (^13^C_1_-containing isotopic exact mass 2164.250 Da). In the same window of retention time, monoisotopic [M-2H]^2-^ peaks of *m/z* 1046.592 and 1114.637, corresponding to heptasaccharide-charged C_60_ and C_70_ DolP, were also observed, albeit at lesser intensities. The same profile also included ^13^C_1_-containing isotopic [M-2H]^2-^ peaks of *m/z* 914.551, 948.575 and 979.583, corresponding to C_60_, C_65_ and C_70_ DolP attached to the pentasaccharide precursor of the complete heptasaccharide, as well as [M-2H]^2-^ peaks of *m/z* 980.585, 1014.089 and 1048.16, corresponding to C_60_, C_65_ and C_70_ DolP attached to the hexasaccharide precursor of the same heptasaccharide (Fig 1A, inset). Note that the *m/z* values reported correspond to either the monoisotopic or ^13^C_1_-containing isotopic peak, depending on which appeared as the highest peak in the mass spectrum of each species.

The heptasaccharide added to sequon-containing peptides in the *in vitro* assay introduced above was shown to comprise a linear pentasaccharide containing N-acetylgalactosamine, two mannoses, glucuronic acid and N-acetylmannosamine, with xyloses attached to the second mannose and the glucuronic acid [18]. To determine whether the same glycan was attached to *P*. *furiosus* DolP in the present study, the monoisotopic [M-2H]^2-^ peak of *m/z* 1081.12 thought to correspond to heptasaccharide-charged C_65_ DolP was further analyzed by tandem mass spectrometry (MS/MS). The fragmentation pattern obtained is consistent with the previously described N-linked glycan (Fig 1B). No sugar stereochemistry is drawn in the chemical structure due to the lack of such information from the MS analysis. Such structural information has, however, been reported by Kohda and co-workers [18].

**Fig 2:**
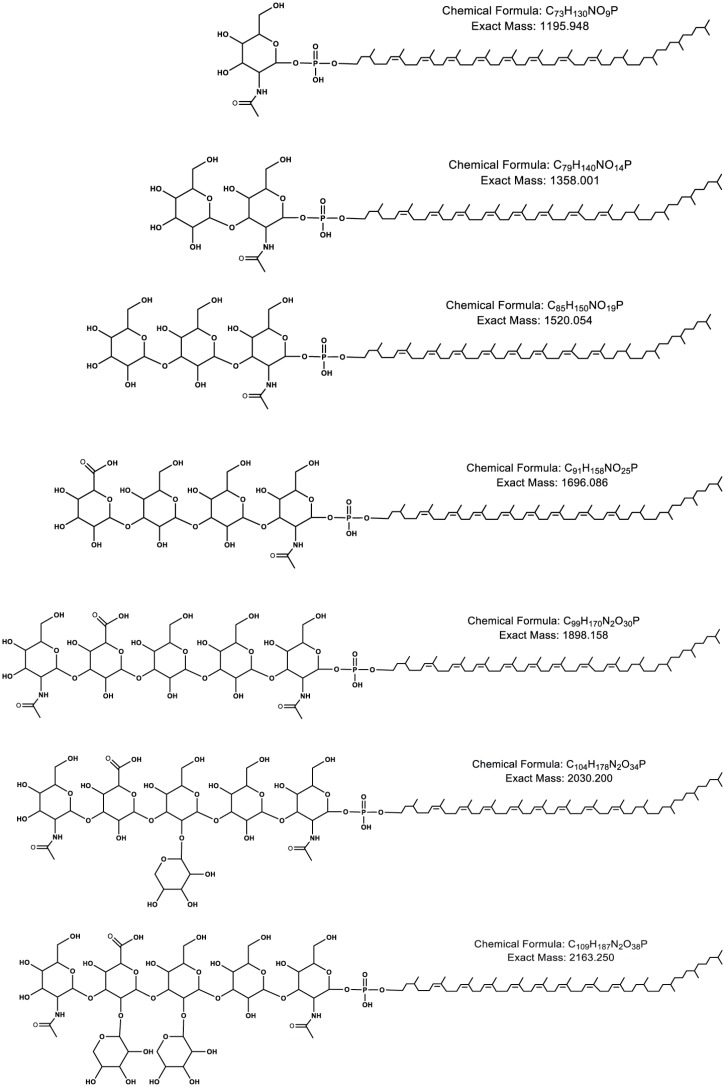
The glycan-charged DolP species detected in the *P*. *furiosus* lipid extract. The predicted chemical structures and calculated masses of C_65_ DolP charged with the mono-, di-, tri-, tetra-, penta-, hexasaccharide-, and the complete heptasaccharide are shown (top to bottom, respectively).

In addition to heptasaccharide-charged DolP, as well as the hexa- and pentasaccharide-charged precursors, RPLC-ESI MS also revealed the presence of tetra-, tri-, di- and monosaccharide-charged DolP in the *P*. *furiosus* lipid extract. The chemical structures and exact masses of different glycan-charged C_65_ DolP species are presented in Fig 2.

Notably, no DolPP-linked glycan species were detected in the total lipid extracts.

### *P*. *furiosus* DolP is highly saturated

**Fig 3:**
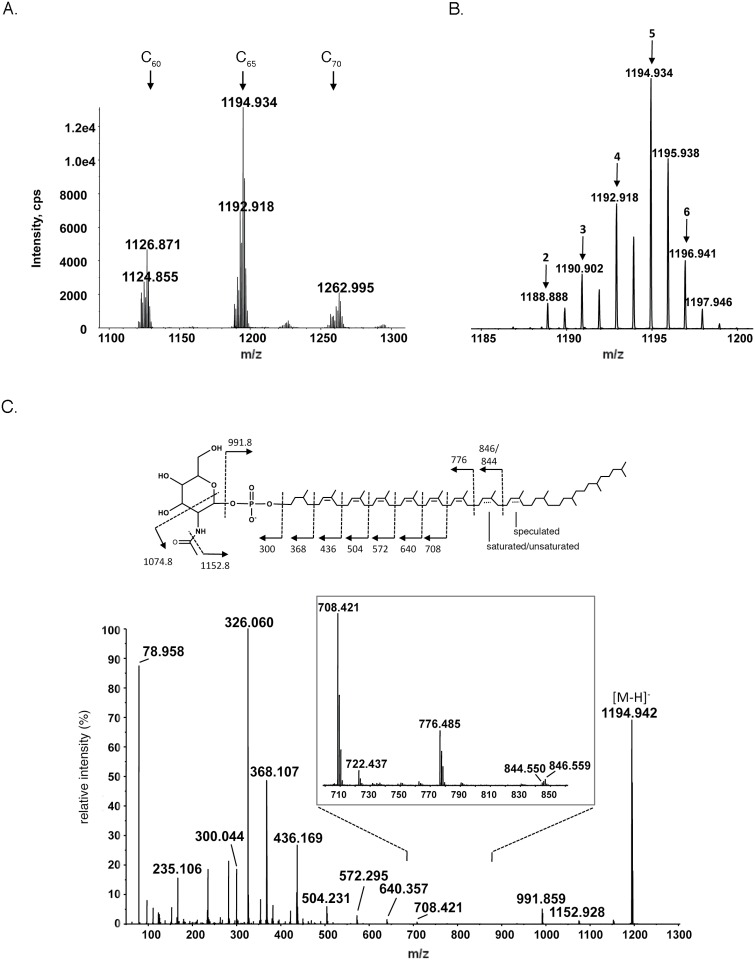
*P*. *furiosus* DolP is highly saturated. A. The MS spectrum of the NPLC fraction with a retention time of 14–14.5 min contains [M-H]^-^ ion peaks corresponding to N-acetylhexosamine-linked C_6o_, C_65_ and C_70_ DolP (*m/z* 1126.871, 1194.934 and 1262.995, respectively). Each DolP species includes 7–11 unsaturated isoprene subunits. B. Zoomed-in spectrum of N-acetylhexosamine-charged C_65_ DolP [M-H]^-^ ion peaks showing the varying degree of dolichol saturation. Peaks corresponding to species containing dolichol that includes 2–6 saturated bonds are indicated accordingly. C. MS/MS spectrum of the [M-H]^-^ ion of C_65_ DolP at *m/z* 1194.934. The fragmentation scheme (inset) shows the distribution of the saturated bonds (mostly near the ω-terminus).

To better characterize the lipid carrier to which the heptasaccharide and its precursors are bound, NPLC-ESI MS was performed. With a retention time of 14–14.5 min, [M-H]^-^ peaks corresponding to monosaccharide (N-acetylhexosamine)-charged C_6o_, C_65_ and C_70_ DolP (*m/z* 1126.871, 1194.934 and 1262.995, respectively) were observed (Fig 3A). Each species comprises a series of DolP variants showing differing degrees of isoprene subunit saturation (Fig 3B). When MS/MS analysis was performed on the [M-H]^-^ ion of C_65_ DolP at *m/z* 1194.934 (Fig 3C), a fragmentation pattern consistent with a DolP molecule presenting a saturated isoprene subunit at the α-position, as well as multiple saturated isoprene subunits close to the ω-end of the polyisoprenoid chain, was obtained The results show that some isoprene units are only partially saturated. For example, the *m/z* 846 and 844 fragments suggest that isoprene-6 (from the ω-end) is only partially saturated. Such heterogeneity makes complete characterization difficult, even with the most advanced MS/MS techniques. Indeed, to the best of our knowledge, no better characterization of dolichols has been previously reported. In addition, a peak at *m/z* 78.950, corresponding to a phosphate group, was noted.

Finally, [M-H]^-^ peaks of *m/z* 983.811, 987.827, 989.844, 991.853, corresponding to C_65_ DolP containing 11, 10, 9 and 8 unsaturated isoprene subunits, respectively, were observed. However, the levels detected were insufficient for MS/MS (not shown).

## Discussion

Across evolution, N-linked glycans are initially assembled on cytoplasmically-oriented phosphorylated polyisoprenoids before delivery to target proteins in the lumen of the endoplasmic reticulum in eukaryotes, in the periplasm in bacteria and on the external surface of the cell in archaea [8,20,21]. Comparison of these lipid carriers, however, reveals domain-specific traits. In bacteria, a nucleotide-charged version of the linking sugar is reacted with UndP to afford an undecaprenol pyrophosphate (UndPP)-linked sugar. Further glycosylation of the resulting UndPP-linked sugar ensues [22]. In higher eukaryotes, a similar process takes place on the cytoplasmic face of the endoplasmic reticulum (ER) membrane and results in the generation of a heptasaccharide-charged DolPP core. Additional individual sugars are subsequently transferred to the DolPP-linked glycan from DolP carriers on the luminal face of the ER membrane [6]. In archaea where lipids involved in N-glycosylation have been studied, it was reported that glycans are attached to DolP carriers [9,11,13,15,19,23,24]. The sole confirmed exception reported to date is *Hbt*. *salinarum*, where one of the two glycans N-linked to the S-layer glycoprotein is derived from a DolPP carrier. Of the 11 Asn residues decorated by glycans in this protein, only Asn-2 bears the unique DolPP-derived glycan; 10 present a common oligosaccharide derived from a DolP carrier [14].

Still, just as archaeal N-linked glycosylation shows diversity in terms of glycan composition and architecture not seen in eukaryotes or bacteria [25], the DolP carrier used in archaeal N-glycosylation also shows considerable variety. Like its eukaryal counterpart, archaeal DolP is saturated at the α-position isoprene but is also saturated at the ω-position isoprene [10,11]. In at least one case, namely *Sulfolobus acidocaldarius*, a thermoacidophilic archaeon that grows optimally at 80°C and pH 2 [26], not only are the α- and ω-isoprenes saturated, so are several internal isoprene units [12]. Indeed, prior to the present study, no other DolPs presenting a similar extent of saturation had been reported. Moreover, archaeal DolPs reported prior to the present report were shown to be shorter than their eukaryal counterparts [27]. In the case of the *S*. *acidocaldarius*, DolP contains only nine isoprene units, many of which are saturated [12]. In this report, it was shown that *P*. *furiosus* DolP isoprenes at both the α- and ω-positions are saturated, as are 1–4 internal isoprenes. At the same time, *P*. *furiosus* DolP is longer than any known archaeal DolP, comprising 12–14 isoprenes, with C_65_ DolP predominating. As such, it would appear that the degree of DolP saturation, but not Dol length, corresponds to an adaptation to life at high temperatures. Indeed, the relative shortness of *S*. *acidocaldarius* DolP (C_45_) could reflect the monolayer nature of its plasma membrane [28]. Moreover, while *S*. *acidocaldarius* belongs to the *Crenarchaeota*, a major archaeal phyla, *P*. *furiosus* is assigned to the *Euryarchaeota*, a second major archaeal phylum [29]. Hence, comparison of phosphorylated dolichols in organisms belonging to these two fundamental branches of the archaeal tree could provide evolutionary insight into the biosynthesis of this molecule.

One possible reason why archaeal N-glycosylation may favor DolP rather than DolPP as the glycan carrier (with *Hbt*. *salinaurum* providing the sole exception to date [14]), may be as an adaptation to the harsh environments archaea can inhabit, since DolPP-glycan derivatives are chemically more labile than the corresponding DolP-glycan derivatives. Indeed, studies addressing the behavior of components of the *Methanococcus voltae* N-glycosylation pathway revealed that sugar-charged DolP but not DolPP served as substrate for AglB and other enzymes [19]. At the same time, computer-based fitting (not shown) revealed that both *P*. *furiosus* DolP-heptasaccharide as characterized in this study and modeled DolPP-heptasaccharide could be accommodated within the solved crystal structure of the *P*. *furiosus* AglB soluble domain [17], implying that steric considerations cannot explain the preferential use of DolP as lipid glycan carrier. Accordingly, *Hbt*. *salinarum* only encodes a single version of AglB that is apparently able to process distinct DolP- and DolPP-bound glycans [30].

Describing the chemical structure of the phosphorylated dolichol purportedly used for N-glycosylation in this organism could potentially be integrated with earlier crystallographic analysis of the C-terminal domain of *P*. *furious* AglB (PF0156) which includes part of the catalytic machinery of this enzyme [17] to provide new structural and mechanistic insight into this protein-processing event. AglB is the archaeal oligosaccharyltransferase, responsible for transferring the phosphorylated dolichol-bound glycan to target protein asparagine residues [31,32]. Although structural information on AglBs from other archaea (i.e. *Archaeoglobus fulgidus* [33–35]) and *Pyrococcus horikoshii* [36]) is available, nothing is yet known of the lipid-linked glycan carrier in these species. To date, the interaction between an oligosaccharyltransferase and the corresponding lipid-linked oligosaccharide substrate has only been proposed based on molecular modeling of the bacterial oligosaccharyltransferase PglB [37].

Finally, DolP modified by the complete heptasaccharide recruited for N-glycosylation in *P*. *furious* [17,18], as well as the hexa- through monosaccharide precursors of this glycan, was detected. As such, it is possible that assembly of the N-linked heptasaccharide on the DolP carrier begins with the sequential addition of the first five heptasaccharide sugars (N-acetylgalactosamine, mannose, mannose, glucuronic acid, N-acetylmannosamine) followed by the addition of xyloses to the second mannose and then to the glucuronic acid. Indeed, glycopeptides modified by the N-linked heptasaccharide as well as by a hexa- and a pentasaccharide respectively lacking one or two xyloses were reported [18]. Still, the techniques used in that study and the present report cannot distinguish between precursors or breakdown products of the hexasaccharide. As such, additional genetic or biochemical studies will be required to delineate the pathway of N-glycosylation in *P*. *furious*.

Further insight into the evolution and biology of archaeal phosphodolichols will be possible as more archaeal genomic sequences are revealed and tools for their manipulation become available, along with the growing number of solved structures of AglB proteins [33–36] and *in vitro* assays for studying the assembly and processing of lipid-linked oligosaccharides [19]. Such efforts could prove important for questions beyond archaeal N-glycosylation.
